# Baricitinib counteracts metaflammation, thus protecting against diet-induced metabolic abnormalities in mice

**DOI:** 10.1016/j.molmet.2020.101009

**Published:** 2020-05-13

**Authors:** Debora Collotta, William Hull, Raffaella Mastrocola, Fausto Chiazza, Alessia Sofia Cento, Catherine Murphy, Roberta Verta, Gustavo Ferreira Alves, Giulia Gaudioso, Francesca Fava, Magdi Yaqoob, Manuela Aragno, Kieran Tuohy, Christoph Thiemermann, Massimo Collino

**Affiliations:** 1 Department of Drug Science and Technology, University of Turin, Turin, Italy; 2 Queen Mary University of London, Center for Translational Medicine and Therapeutics, William Harvey Research Institute, Barts and the London School of Medicine and Dentistry, London, UK; 3 Department of Clinical and Biological Sciences, University of Turin, Turin, Italy; 4 Edmund Mach Foundation, San Michele all'Adige, Italy

**Keywords:** Metaflammation, Baricitinib, High-fat-high sugar diet, JAK2-STAT2 pathway, Insulin resistance

## Abstract

**Objective:**

Recent evidence suggests the substantial pathogenic role of the Janus kinase (JAK)/signal transducer and activator of transcription (STAT) pathway in the development of low-grade chronic inflammatory response, known as “metaflammation,” which contributes to obesity and type 2 diabetes. In this study, we investigated the effects of the JAK1/2 inhibitor baricitinib, recently approved for the treatment of rheumatoid arthritis, in a murine high-fat-high sugar diet model.

**Methods:**

Male C57BL/6 mice were fed with a control normal diet (ND) or a high-fat-high sugar diet (HD) for 22 weeks. A sub-group of HD fed mice was treated with baricitinib (10 mg/kg die, p.o.) for the last 16 weeks (HD + Bar).

**Results:**

HD feeding resulted in obesity, insulin-resistance, hypercholesterolemia and alterations in gut microbial composition. The metabolic abnormalities were dramatically reduced by chronic baricitinib administration. Treatment of HD mice with baricitinib did not change the diet-induced alterations in the gut, but restored insulin signaling in the liver and skeletal muscle, resulting in improvements of diet-induced myosteatosis, mesangial expansion and associated proteinuria. The skeletal muscle and renal protection were due to inhibition of the local JAK2-STAT2 pathway by baricitinib. We also demonstrated that restored tissue levels of JAK2-STAT2 activity were associated with a significant reduction in cytokine levels in the blood.

**Conclusions:**

In summary, our data suggest that the JAK2-STAT2 pathway may represent a novel candidate for the treatment of diet-related metabolic derangements, with the potential for EMA- and FDA-approved JAK inhibitors to be repurposed for the treatment of type 2 diabetes and/or its complications.

## Highlights

• The Jak/Stat signaling pathway is dysregulated in metabolic diseases.
• The Jak2 inhibitor baricitinib exerts multi-organ protection against diet-induced metabolic derangements.
• Targeting the chronic metabolic inflammation (termed metaflammation) represents an effective strategy for metabolic diseases.

## 1 Introduction

Low-grade, chronic inflammatory response, known as “metaflammation,” exerts a key role in promoting diet-related metabolic disorders [1]. Several studies have provided clear evidence of a causative relationship between exposure to hypercaloric diets and quantitative and qualitative changes in intestinal commensal bacteria communities, which can, in turn, lead to metabolic endotoxemia and the over-production of inflammatory mediators, which drive the development of dysglycemic and dyslipidemic states [2]. Treatments that halt or induce regression of metaflammation have the potential to provide immense clinical, social and economic benefits. However, only limited research is available regarding the identification of key inflammatory pathways activated by the metabolic, biochemical and hemodynamic derangements known to exist in metabolic diseases. The Janus kinase (JAK) signal transducer and activator of transcription (STAT) pathway has recently been described as a potential master regulator of signaling events, regulating more than 50 cytokine or hormone receptors, many of which play pivotal roles in the pathophysiology of metaflammation [3,4]. JAK1, JAK2, JAK3 and tyrosine kinase 2 (TYK2) are different isoforms of the mammalian JAK family [5]. They can be selectively activated by different receptors, leading to specific or relatively discrete functional outcomes. JAK signaling has been demonstrated to be dysregulated in metabolic diseases including obesity and type 2 diabetes mellitus (T2DM) [6]. Studies of knock-out mice confirmed the pivotal role of JAK signaling, mainly JAK2, in the regulation of a multitude of metabolic processes including, but not limited to, glucose tolerance, insulin sensitivity, leptin sensitivity, energy expenditure and adiposity. Clinically suitable STAT inhibitors are not yet available, whereas small molecule inhibitors of the upstream JAK proteins have been recently developed and are now approved for several chronic inflammatory disorders including rheumatoid arthritis, ulcerative colitis, myelofibrosis and polycythemia. The efficacy of these drugs is thought to be due to reduced T and B cell activation [7] and therefore a reduction in the inflammation that drives the progression of the disease itself [8,9]. However, to the best of our knowledge, the effects of the pharmacological modulation of JAK signaling in the clinical context of insulin resistance and diet-related metabolic disorders have been poorly investigated. A recently published study on rats with streptozotocin-induced diabetes investigated the effects of the pharmacological inhibition of JAK signaling. The authors demonstrated that combination therapy of tofacitinib (10 mg/kg BW) and aspirin (100 mg/kg BW) in rats significantly improved glucose homeostasis and insulin secretion compared to diabetic (untreated) rats [10]. Tofacitinib is a JAK3 inhibitor. Other authors have convincingly demonstrated that loss of JAK3 in mice results in body weight gain, impaired glycemic and lipid homeostasis and early symptoms of liver steatosis [11]. In contrast, JAK2 deficiency in the liver, macrophages, or adipocytes protects against high-fat diet induced metabolic inflammation [[12], [13], [14]], suggesting that JAK2 may be a promising target for the treatment of obesity, metabolic syndrome and T2DM. The JAK1/2 inhibitor baricitinib was approved in February 2017 for the treatment of moderate to severe active rheumatoid arthritis. Baricitinib shows a selective and balanced potency against JAK1 (IC_50_ 5.9 nM) and JAK2 (IC_50_ 5.7 nM) with far less potency for JAK3 (IC_50_ > 400 nM), thus being considered a JAK3 sparing agent. In a recently concluded phase 2, double-blind, dose-ranging study in patients with T2DM and diabetic kidney disease, baricitinib was demonstrated to decrease inflammatory biomarkers over 24 weeks and this effect was associated with a significant reduction in albuminuria [15]. In this study, we investigated the effects of the pharmacological modulation of the JAK cascade with baricitinib in an *in* *vivo* model of diet-induced metabolic alterations to evaluate the efficacy and mechanism of action of baricitinib to provide “proof of concept” evidence for the repurposing of JAK inhibitors in metabolic diseases.

## 2 Materials and methods

### 2.1 Animals and experimental procedures

The *in* *vivo* experimental procedures described herein were approved by the local Animal Use and Care Committee and the Ministry of Health (approval no. 42/2017-PR) in keeping with the European Directive 2010/63/EU on the protection of animals used for scientific purposes as well as the Guide for the Care and Use of Laboratory Animals. This study was conducted using 4-week-old male C57BL/6 mice maintained in conventional housing conditions in a controlled environment at 25 ± 2 °C. The mice were co-housed one week prior to the onset of the experiments and randomly allocated to three experimental groups (n = 15 per group): mice fed a control normal diet (ND group), mice fed a high-fat and high-sugar diet (45 kJ% fat, 35 kJ% sugar) for 22 weeks (HD group), and mice fed an HD for 22 weeks and treated with baricitinib (10 mg/kg die, p.o.) for the last 16 weeks (HD + Bar). The dietary protocol chosen was based on those used in previous animal studies showing that similar compositions and kinetics of dietary manipulation resulted in robust changes in lipid and glucose profiles as well as body weight gain [[16], [17], [18]]. Body weight and food/water intake were recorded weekly, whereas fasting glucose was recorded monthly. Urine and feces samples were collected at weeks 0, 5 and 22 (18 h metabolic cages). Total urinary protein and urine albumin concentrations were compared with creatinine concentrations to calculate the albumin to creatinine ratio (ACR) as an indicator of albuminuria. Baricitinib was administered as an added dietary component. The chronic administration of the baricitinib dose used in this study did not lead to adverse effects [19] and has been shown to reduce the contribution of Th1 cells to metaflammation in obese mice [20].

### 2.2 Oral glucose tolerance test (OGTT)

One day before the end of the experiment, an OGTT was conducted after an overnight fasting period. Glucose (2 g/kg) was administered by oral gavage and blood was obtained from the saphenous vein once before the glucose administration and after 15, 30, 60 and 120 min. The glucose concentration was measured with a conventional glucometer (GlucoMen LX kit, Menarini Diagnostics, Grassina, Italy).

#### 2.2.1 MRI

The mice were anesthetized using 5% isoflurane and placed on a bed with water heated to 50 °C passing through it to maintain body temperature. Respiration was monitored using a pressure sensor placed under the abdomen. The mice were imaged in a Brucker ICON 1T preclinical MRI scanner using a body coil and a T2 weighted RARE 3D isotropic image with TR of 1500 ms and TE of 84 ms and a voxel size of 0.219 X 0.375 × 0.375 mm. Images were analyzed using VivoQuant software (Invicro LLC, Boston, MA, USA). 3D regions of interest (ROI) were used to isolate the quadriceps muscle of a randomly selected mouse. Within the ROI, a threshold was set for all of the pixels appearing to contain fat and the pixel volume was quantified by the software. The results for both of these volumes are presented as a percentage of the total quad area exceeding the fat threshold.

### 2.3 Biochemical analysis

After 22 weeks of dietary manipulation, the mice (fasted for 4 h) were anesthetized using isoflurane (IsoFlo, Abbott Laboratories) and killed by cardiac exsanguination. Blood samples were collected and the plasma was isolated. Plasmatic concentrations of triglycerides (TGs), total cholesterol, high-density lipoprotein (HDL) and low-density lipoprotein (LDL) were measured using reagent kits according to standard enzymatic procedures (Hospitex Diagnostics, Florence, Italy). Plasma insulin, leptin, ghrelin, glucose-dependent insulinotropic polypeptide (GIP), glucagon-like peptide (GLP-1), resistin, IL-1β, TNF-α, IFN-ɣ, IL-6 and IL-10 levels were measured using a Luminex suspension bead-based multiplexed Bio-Plex 3D system (Bio-Rad Laboratories, Hercules, CA, USA).

### 2.4 Fecal microbiota analysis

Total genomic DNA extraction from frozen feces was conducted using a QIAamp PowerFecal DNA Isolation kit (MO BIO Laboratories, Inc., Carlsbad, CA, USA) and then subjected to PCR amplification by targeting 16S rRNA V3–V4 variable regions with specific bacterial primer set 341F (5′-CCTACGGGNGGCWGCAG-3′) and 806R (5′-GACTACNVGGGTWTCTAATCC-3′) as previously reported [21]. PCR products were assessed via gel electrophoresis and cleaned using an Agencourt AMPure XP system (Beckman Coulter, Brea, CA, USA) following the manufacturer's instructions. After 7 PCR cycles (16S Metagenomic Sequencing Library Preparation, Illumina), Illumina adaptors were attached (Illumina Nextera XT Index Primer). Libraries were purified using Agencourt AMPure XP (Beckman) and then sequenced on an Illumina MiSeq (PE300) platform (MiSeq Control software 2.0.5 and Real-Time Analysis software 1.16.18). Sequences obtained from Illumina sequencing were analyzed using the Quantitative Insights Into Microbial Ecology (QIIME) 2.0 pipeline [22]. The percentage relative abundance of taxa from different dietary groups were compared using the non-parametric Wilcoxon statistical test.

### 2.5 Tissue extracts

Liver, skeletal muscle and kidney extracts were prepared as previously described [23]. Briefly, tissues were homogenized and centrifuged at 10,000 × *g* for 20 min at 4 °C. Supernatants were collected and the protein content was determined using a BCA protein assay (Pierce Biotechnology Inc., Rockford, IL, USA) following the manufacturer's instructions.

### 2.6 Skeletal muscle TG level

TGs were extracted from the total tissue homogenates of the skeletal muscle and assayed using a triglyceride quantification kit (Abnova Corporation, Aachen, Germany).

### 2.7 Western blotting analysis

Proteins were separated by 8% sodium dodecyl sulphate-polyacrylamide gel electrophoresis (SDS-PAGE) and transferred to a polyvinyldenedifluoride (PVDF) membrane, which was then incubated with primary antibodies (dilution 1:1000). The antibodies used were rabbit anti-Tyr^1007/1008^ JAK2 (#3776), rabbit anti-total JAK2 (#3230), rabbit anti-Tyr^690^ STAT2 (Bioss Antibodies, bs-3428R), rabbit anti-total STAT2 (#72604), rabbit p21 (#2947), rabbit anti-Ser^307^ IRS-1 (#2381), mouse anti-total IRS-1 (#3194), rabbit anti-Ser^473^ AKT (#4060), rabbit anti-total AKT (#9272), rabbit anti-Ser^9^ GSK-3β (#9332) and rabbit anti-total GSK-3β (9315).

The blots were then incubated with secondary antibodies conjugated with horseradish peroxidase (dilution 1:20000) and developed using an ECL detection system. Bio-Rad Image Lab Software 6.0.1 was used to analyze the immunoreactive bands and the results were normalized to the controls.

### 2.8 Kidney histopathological examination

Histology was conducted on formalin-fixed paraffin-embedded (FFPE) samples that were subsequently stained with either hematoxylin and eosin (H&E stain) or periodic acid Schiff stain (PAS) before being visualized using a NanoZoomer digital pathology scanner (Hamamatsu Photonics K.K., Hamamatsu City, Japan). The samples were then analyzed using a NanoZoomer NDP viewer software (Hamamatsu Photonics K.K., Japan). Two cross-sectional measurements (at 90° to one another) were obtained for 10 renal corpuscles and glomeruli and averaged before an area for each was established using the formula A = πr2.

### 2.9 Materials

Unless otherwise stated, all of the compounds were purchased from Sigma–Aldrich Ltd. (St. Louis, MO, USA). The BCA Protein Assay kit was obtained from Pierce Biotechnology Inc. (Rockford, IL, USA). Antibodies were purchased from Cell-Signaling Technology (Beverly, MA, USA).

### 2.10 Statistical analysis

All of the values in the text and figures are expressed as mean ± S.E.M. for *n* observations. The data distribution were verified by the Shapiro–Wilk normality test and the homogeneity of variances by the Bartlett test. Normally distributed data were assessed by one-way ANOVA followed by the Newman–Keuls post hoc test. Data that were not normally distributed were analyzed with the Kruskal–Wallis test followed by Dunn's test. OGTTs were analyzed using the area under the receiver operating characteristic (ROC) curve. A p value < 0.05 was considered statistically significant. Statistical analysis was conducted using GraphPad Prism 5.03 (GraphPad Software, San Diego, CA, USA).

## 3 Results

### 3.1 Effects of dietary manipulation and baricitinib administration on metabolic parameters and gut microbiota composition

**Figure 1:**
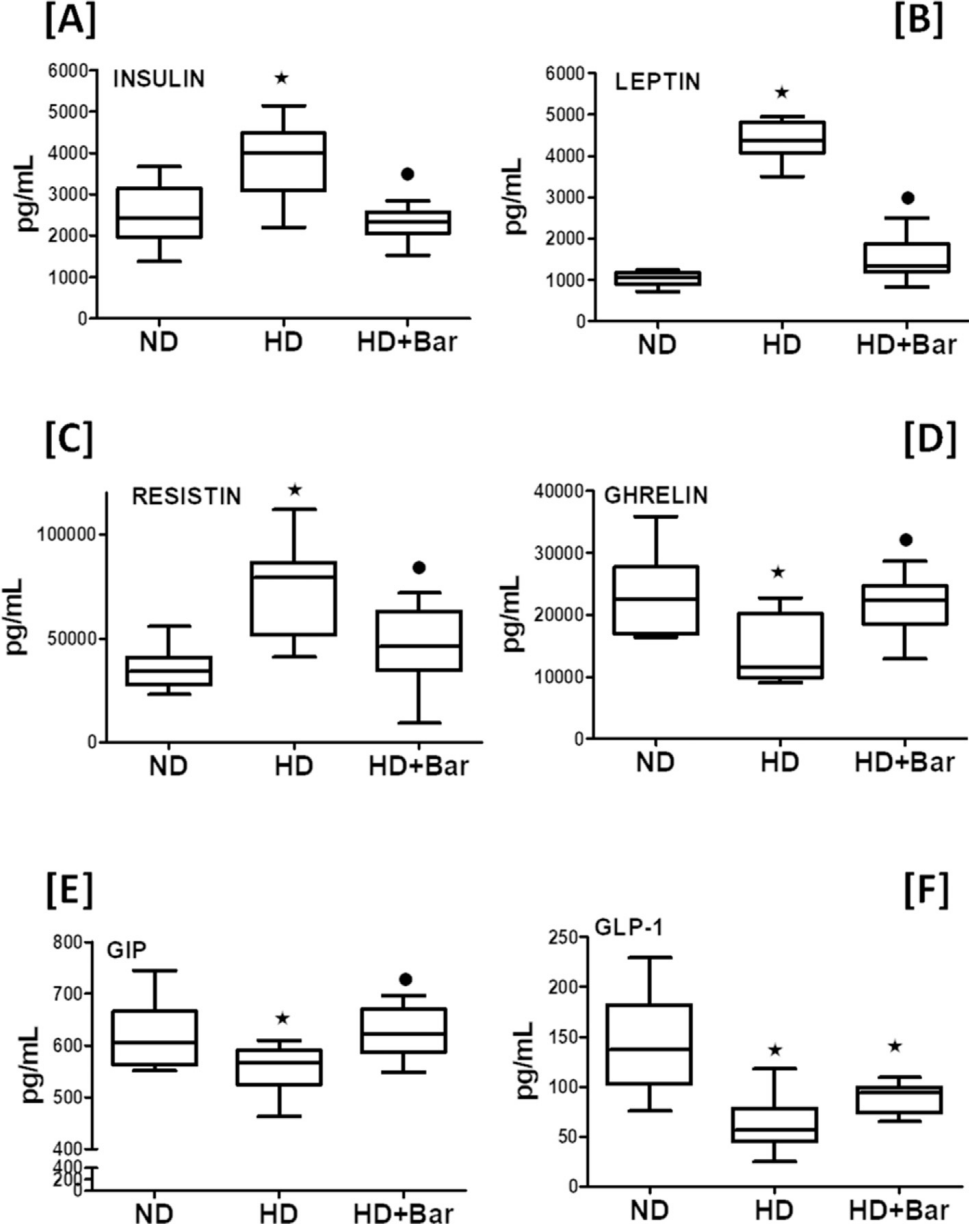
Plasma concentrations of: insulin (A), leptin (B), resistin (C), ghrelin (D); GIP (E), and GLP-1 (F). All of the data are expressed as mean ± SEM for n = 15 per group. ∗*p*<0.05 *vs* ND and •*p*<0.05 *vs* HD.

**Figure 2:**
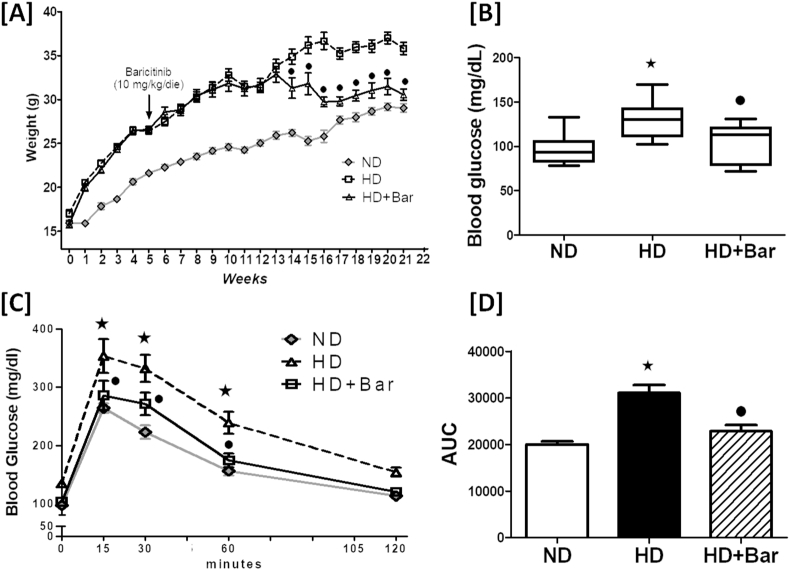
Effects of diet and baricitinib on mouse body weight (A), basal non-fasted blood glucose and oral glucose tolerance at 22 weeks (B and C). Panel D shows the area under curve (AUC) of the oral glucose tolerance test calculated for respective groups (n = 6 per group) and used for statistical analysis (D). All of the data are expressed as mean ± SEM for n number of observations. ∗*p*<0.05 *vs* ND and •*p*<0.05 *vs* HD.

When compared to the ND fed mice, the mice fed an HD had elevated plasma levels of insulin, leptin,and resistin (Figure 1A–C) associated with decreased plasma levels of ghrelin, GIP and GLP-1 (Figure 1D–F). Treatment of the mice fed a hypercaloric diet with baricitinib (HD + Bar) restored these master hormonal regulators of metabolism back to values similar to those seen in the control mice, but the effect of baricitinib on GLP-1 was not significant. Although no significant differences in food intake (g/die) among any of the experimental groups were recorded, the mice exposed to the hypercaloric diet showed a significant increase in daily caloric intake (14.83 ± 1.63 kcal/die) compared to the mice fed the normocaloric diet (8.30 ± 0.18 kcal/die), resulting in weight gain (Figure 2A), higher blood glucose levels (Figure 2B), and OGTT impairment (Figure 2C,D). The diet changes in the body weight and blood glucose levels were significantly reduced in the HD fed mice treated with baricitinib, which did not significantly affect either food or calorie intake.

**Figure 3:**
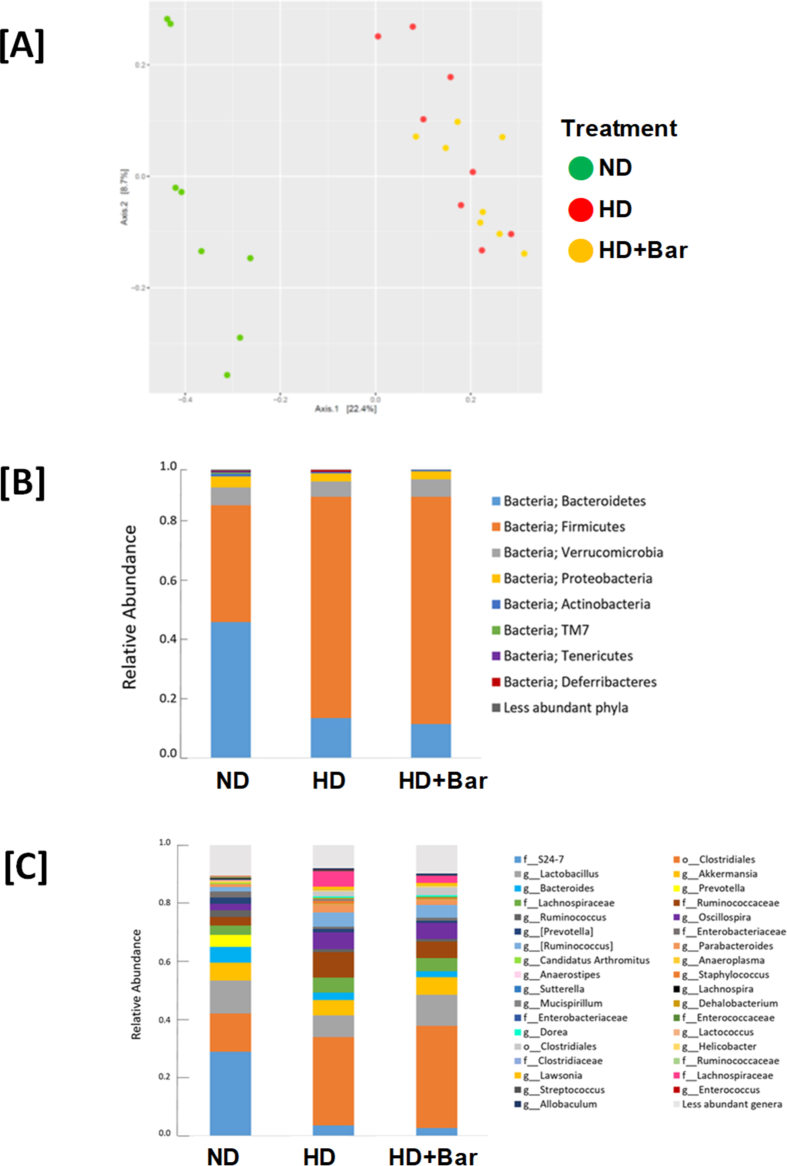
PCoA showing differences in beta-diversity at T22 (Bray–Curtis dissimilarity index). The relative abundance in the 3 dietary groups at the phylum (A) and genus (B) levels.

No differences were observed in the composition of the microbiota at T0 (baseline) among any of the groups studied. The HD and HD + Bar mice significantly differed from the ND mice in fecal microbial β diversity at T11 (not shown) and T22 weeks (Figure 3A), but not in α diversity (not shown). At the phylum level, the ND mice had higher Bacteroidetes/Firmicutes ratios at T22 than the HD (p = 0.001) and HD + Bar (p = 0.0008) mice. Both the HD and HD + Bar mice differed from the ND mice for decreased *Bacteroides* (p = 0.027 and p = 0.04, respectively), *Ruminococcus* (p < 0.001 and p = 0.003, respectively), *Anaerostipes* (p < 0.001 and p = 0.001, respectively), *Anaeroplasma* (p < 0.001 and p = 0.001, respectively), and Muribaculaceae family (p < 0.001 and p = 0.001, respectively) and increased *Dehalobacterium* (p = 0.018; p = 0.05, respectively), *Lachnospira* (p < 0.001 and p = 0.005, respectively), *Dorea* (p < 0.001 and p = 0.003, respectively), *Oscillospira* (p = 0.002 and p = 0.003, respectively), *Streptococcus* (p < 0.001 and p = 0.004, respectively), *Lawsonia* (p < 0.001 and p = 0.003, respectively), and Lachnospiraceae family (p < 0.001 and p = 0.002, respectively), indicative of high-sugar, high-fat, and diet-induced microbiota imbalances (Figure 3B,C). Moreover, the HD mice treated with baricitinib also differed from the ND mice for decreased *Prevotella* (p = 0.002).

### 3.2 Baricitinib ameliorates local insulin signal transduction affected by HD

**Figure 4:**
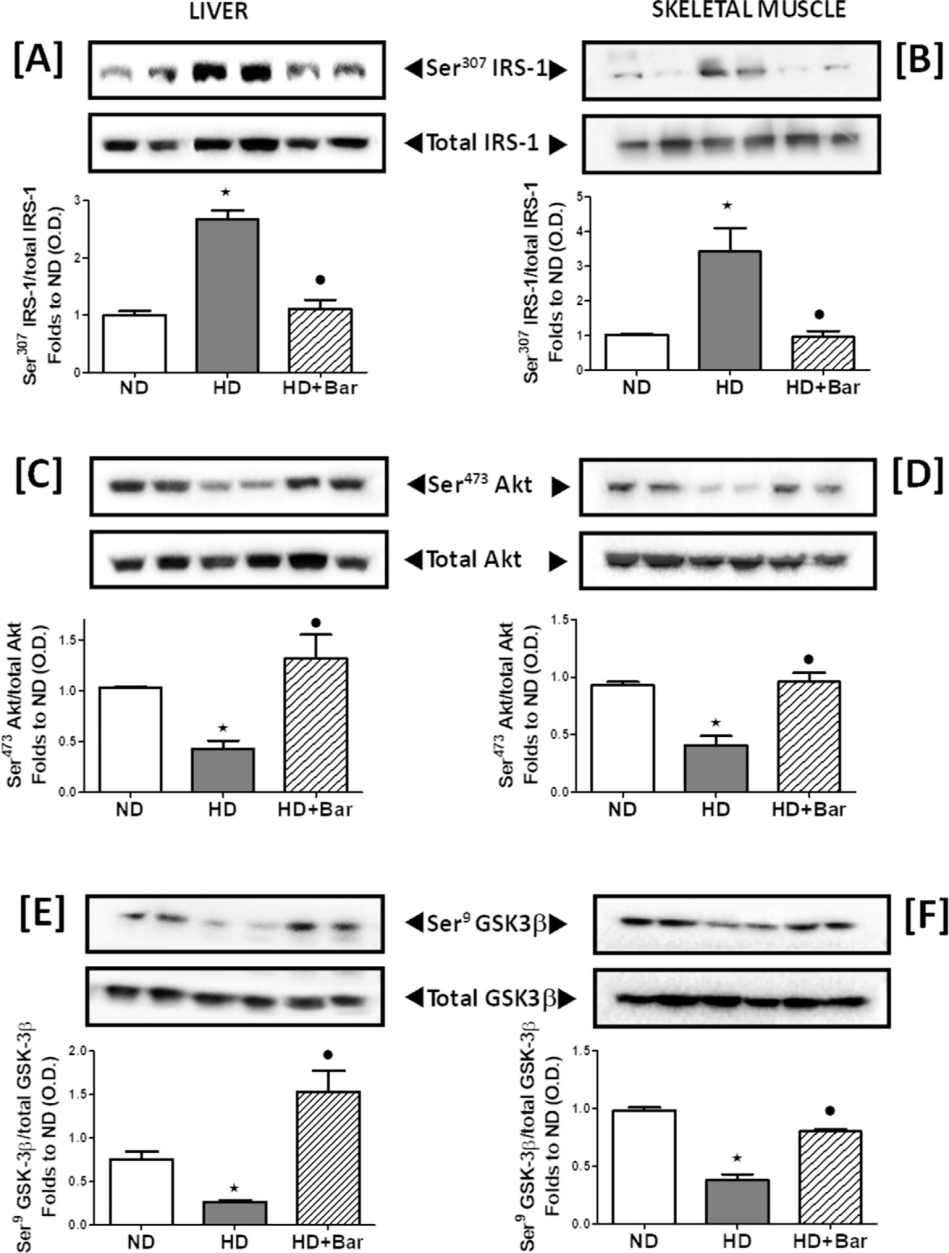
Effects of dietary manipulation and baricitinib administration on the insulin signaling pathway. Western blotting analysis showing phosphorylation of Ser^307^ on IRS-1 in the liver (A) and skeletal muscle (B), Ser^473^ on AKT in the liver (C) and skeletal muscle (D), and Ser^9^ on GSK-3β in the liver (E) and skeletal muscle (F) normalized to the related total forms. All of the data are expressed as mean ± SEM for n = 6 per group. ∗ p<0.05 *vs* ND and •p< 0.05 *vs* HD.

Chronic exposure to HD was associated with a robust increase in Ser^307^ phosphorylation of insulin receptor substrate-1 (IRS-1) in liver (Figure 4A) and skeletal muscle (Figure 4B) compared to ND feeding. These effects were associated with a robust hepatic and skeletal muscle decrease in Ser^473^ phosphorylation of protein kinase B (AKT) (Figure 4C,D) and Ser^9^ phosphorylation of glycogen synthase kinase-3β (GSK-3β) (Figure 4E,F), two of the main downstream mediators of the insulin signaling pathway. These alterations in protein phosphorylation were suggestive of changes in the activation status of the respective proteins, thus indicating that HD led to impairments in insulin signaling. Interestingly, treatment of the HD mice with baricitinib counteracted the changes in insulin signaling evoked by HD, highlighting the drug's ability to counteract diet-induced impairments in the insulin signal transduction pathway.

### 3.3 Baricitinib attenuates dyslipidemia and myosteatosis

**Figure 5:**
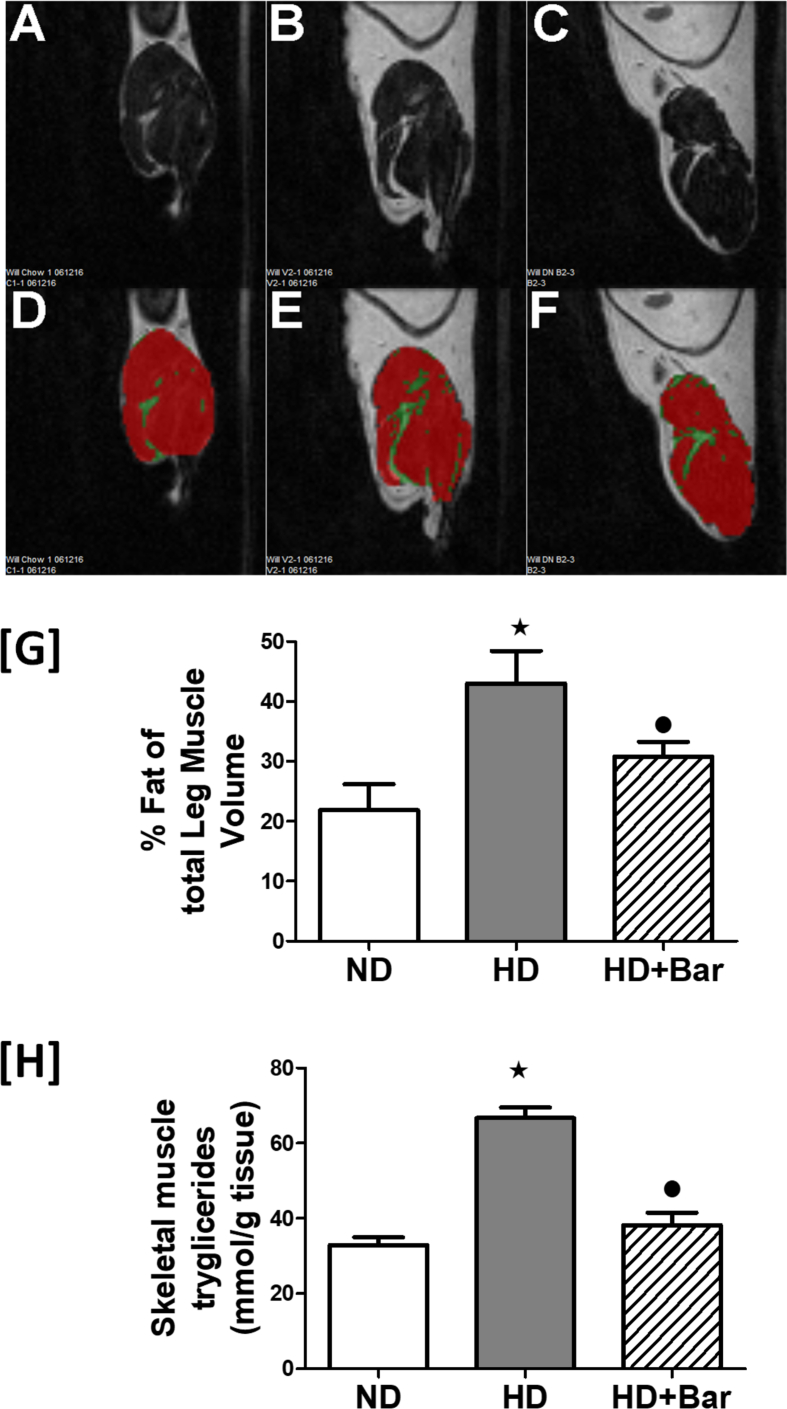
MRI analysis of the quadriceps muscle (A, B, C, D, E and F). Effects of HD and baricitinib administration on fat deposition in the skeletal muscle (G) and skeletal muscle triglyceride levels (H). All of the data are expressed as mean ± SEM for n = 6 per group. ∗*p*< 0.05 *vs* ND and •*p*< 0.05 *vs* HD.

The mice exposed to HD showed marked increases in their plasma serum triglyceride levels (1.07 ± 0.02 mmol/L) and total cholesterol (3.43 ± 0.07 mmol/L) and LDL/HDL ratios (0.43 ± 0.03) compared to the control mice (0.87 ± 0.03, 2.77 ± 0.07, and 0.32 ± 0.04, respectively). The diet-induced changes in the plasma lipid profiles were attenuated by treatment of the HD mice with baricitinib (0.98 ± 0.03, 2.89 ± 0.03, and 0.32 ± 0.02, respectively). The systemic alterations in the lipid profiles evoked by HD were associated with a significant deposition of fat within the skeletal muscle as shown by MRI analysis of the quadriceps muscle and confirmed by a two-fold increase in the skeletal muscle triglyceride levels. In contrast, treatment of the HD mice with baricitinib significantly attenuated the increase in fat deposition and triglyceride content in the skeletal muscle (Figure 5).

### 3.4 Baricitinib attenuates the HD-induced renal damage

**Figure 6:**
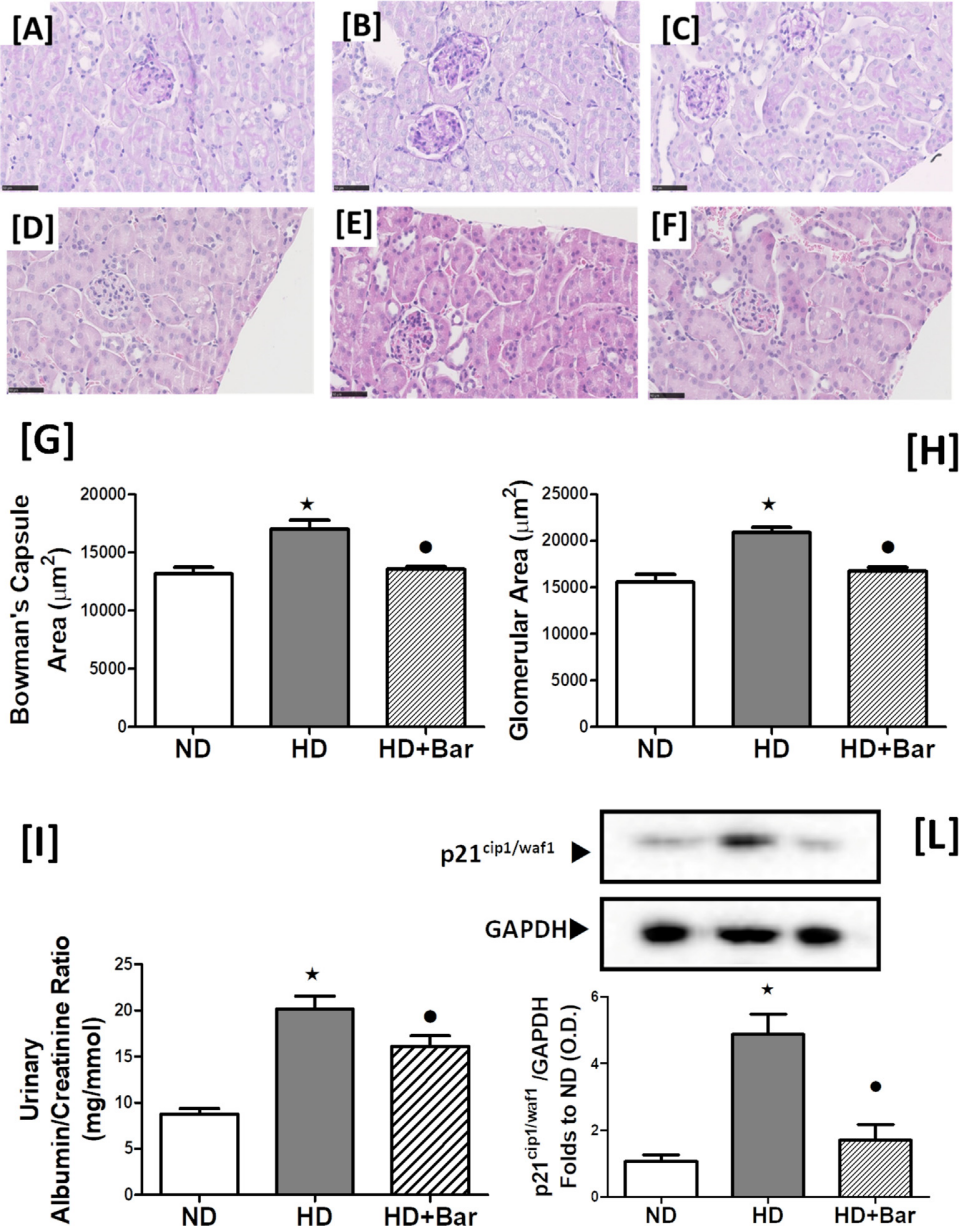
Effects of HD and baricitinib administration on the kidneys. Acid-Schiff positive staining within the glomeruli (an indicator of mesangial expansion) (A, B and C) and histological analysis of kidney sections (D, Eand F). Bowman's capsule area (G), glomerular area (H), urinary albumin creatinine ratios (ACR; I), and expression level of cyclin kinase inhibitor (p21, L) were evaluated in the kidneys of the mice exposed to ND or HD in the absence or presence of baricitinib (10 mg/kg die, p.o.). Values are mean ± SEM for n = 6 per group. ∗*p*<0.05 *vs* ND and •*p*<0.05 *vs* HD.

As shown in Figure 6, the mice fed an HD showed a significant increase in their urinary albumin creatinine ratio (ACR) in comparison to mice fed ND, indicating the development of proteinuria, which was reduced by baricitinib administration (Figure 6I). As proteinuria is often associated with dilatations of Bowman's capsule and glomerular hypertrophy, we conducted a histological analysis of the kidneys obtained from all of the animals (Figure 6A–F). The average size of the glomeruli in the HD mice was larger than in the ND mice, indicating glomerular hypertrophy (Figure 6H). This effect was associated with an increase in the renal corpuscle area (Figure 6G). In contrast, treatment of the mice fed an HD with baricitinib abolished the glomerular hypertrophy and increased Bowman's space. A profound increase in periodic acid-Schiff positive staining within the glomeruli (an indicator of mesangial expansion) was recorded in the kidneys of the HD mice compared to the ND mice (Figure 6A,B). In contrast, treatment of the HD mice with baricitinib abolished the increase in mesangial expansion (Figure 6C). A marker of mesangial expansion that has been demonstrated to be increased in different types of glomerular diseases is the cell cycle activator p21 (cyclin-dependent kinase inhibitor), the upregulation of which is a direct consequence of the activation of the JAK-STAT pathway [24]. Compared to the mice fed a normal chow diet, the mice fed an HD showed a significant increase in the p21 levels in the kidneys (Figure 6L), suggesting an early stage of diabetic nephropathy. This increase in p21 was largely attenuated in the mice fed an HD and treated with baricitinib.

### 3.5 Baricitinib reduces local JAK2 activity and systemic inflammatory response

**Figure 7:**
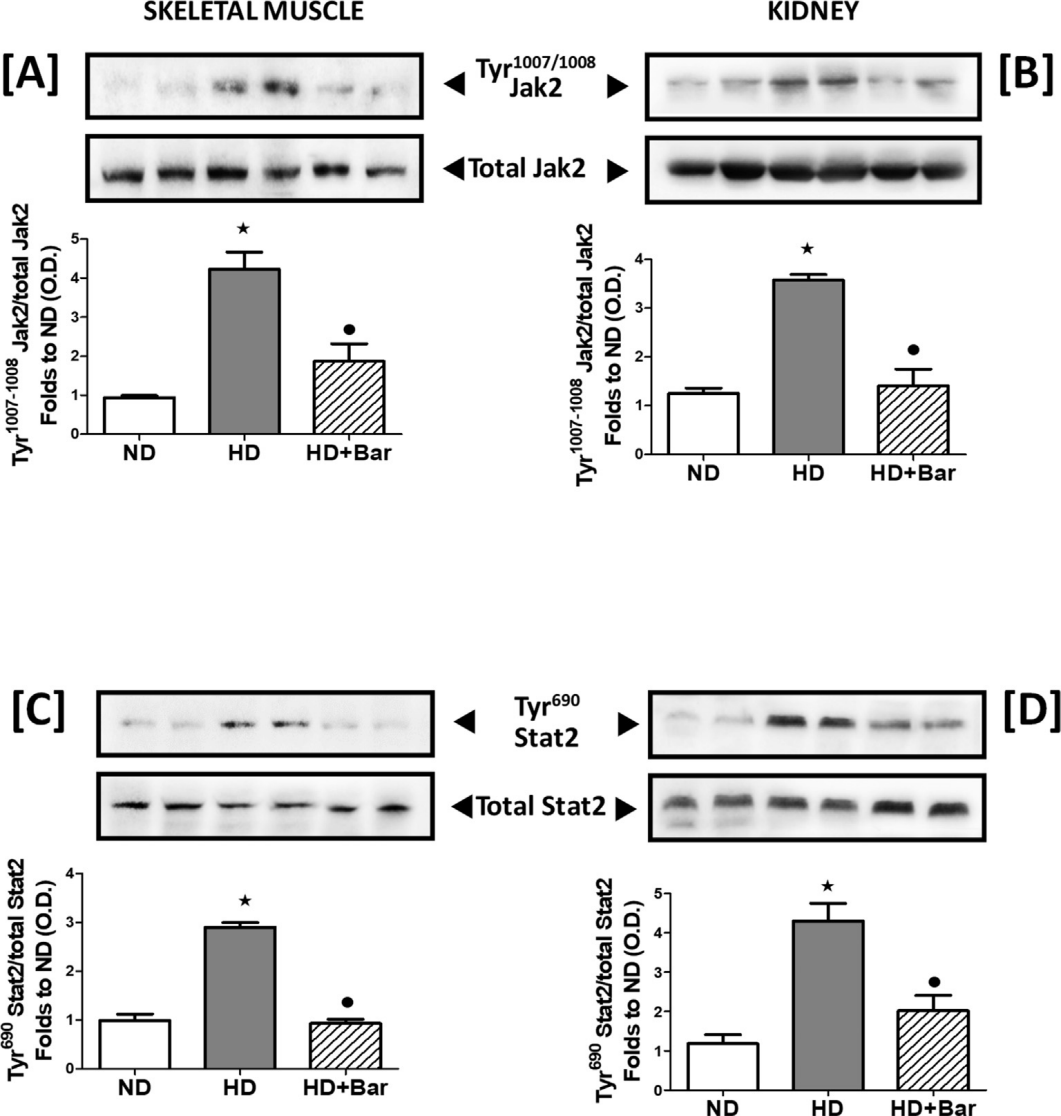
Baricitinib attenuates the HD-induced JAK-STAT pathway. Western blotting analysis for phosphorylation of Tyr ^1007/1008^ JAK1/2 in the skeletal muscle (A) and kidneys (B) and normalized to total JAK1/2 and for phosphorylation of Tyr ^690^ on STAT2 in the skeletal muscle (C) and kidneys (D) and normalized to total STAT2. All of the data are expressed as mean ± SEM for n = 6 per group. ∗p< 0.05 *vs* ND and •p< 0.05 *vs* HD.

**Figure 8:**
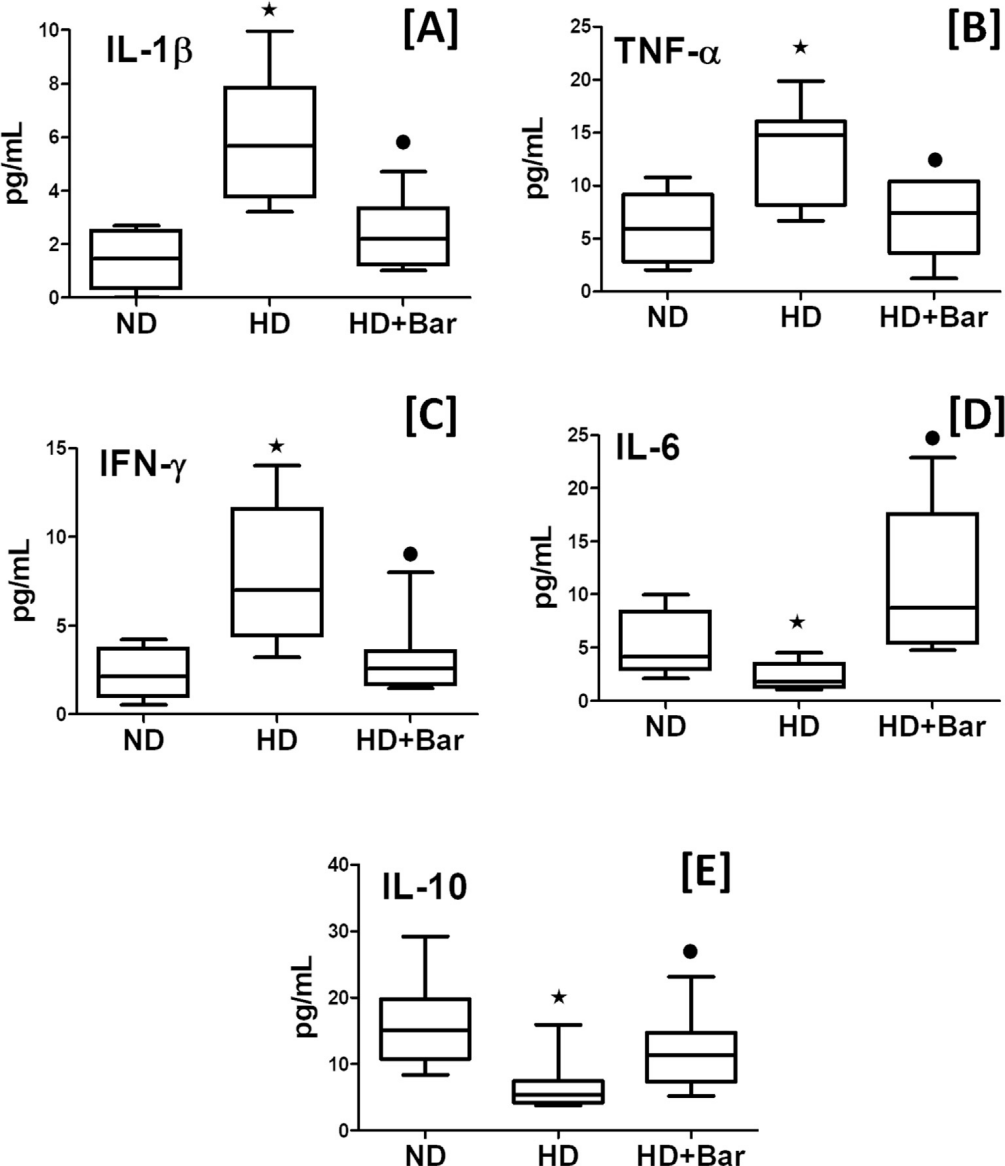
Plasma concentration of inflammatory cytokines Il-1β (A), TNF-α (B), IFN (C), IL-6 (D), and IL-10 (E). All of the data are expressed as mean ± SEM for n = 15 per group. ∗*p*<0.05 *vs* ND and •*p*<0.05 *vs* HD.

As shown in Figure 7 the skeletal muscle and kidney tissues of the mice fed an HD exhibited a significant increase in the phosphorylation of Tyr^1007/1008^ on JAK2 (Figure 7A–B), suggesting increased activation resulting in a downstream increase in Tyr^690^ phosphorylation of STAT2 (Figure 7C–D), demonstrating that the JAK2-STAT2 signaling pathway was activated in the skeletal muscle and kidneys of the mice that demonstrated metabolic derangement. Taken together, these results show that diet-induced increase in tissue dysfunction and injury are associated with over-activation of the pro-inflammatory JAK2-STAT2 pathway. In contrast, the mice fed an HD and treated with baricitinib had significantly reduced activation of JAK2 and downstream effector STAT2, confirming baricitinib's ability to interfere with its pharmacological target in our experimental conditions. The local over-activation of JAK2-STAT2 was paralleled by increased plasma levels of the pro-inflammatory mediators IL-1β, TNFα, and IFN-γ as well as reduced levels of anti inflammatory mediators IL-6 and IL-10 (Figure 8). Interestingly, the systemic levels of pro-inflammatory cytokines that signaled through the JAK-STAT pathway, such as IL-1β, TNFα, and IFN-γ [[25], [26], [27]], were reduced in the plasma of the HD mice exposed to baricitinib. Baricitinib administration was simultaneously associated with increased blood concentrations of IL-6 and IL-10, suggesting that local inhibition of JAK2-STAT2 contributes to reducing the severity of systemic metaflammation.

## 4 Discussion

Unhealthy dietary habits along with sedentary behavior trigger a multitude of pathophysiological changes evoking chronic metabolic inflammation (called metaflammation), ultimately leading to the development of obesity and insulin resistance [28]. In this study, we confirmed that the mice exposed to HD for 22 weeks showed an excessive production of pro-inflammatory cytokines accompanied by the development of the expected dysmetabolic phenotype with increased body weight, adiposity and elevated circulating insulin, glucose and lipid levels. We also demonstrated that the hypercaloric diet reshaped the gut microbial composition, inducing an obese microbiota phenotype, depleting Bacteroidetes (Muribaculaceae and *Bacteroides*) and increasing *Ruminococcus*, *Oscillospira*, *Dorea* and the Lachnospiraceae family [29,30]. Consistent with previous studies, this microbial composition can be induced by either obesogenic diets or genetic mutations that lead to insulin resistance, inflammation and excessive weight gain [[31], [32], [33], [34]]. Our results showed that the gut microbiota of both the HD and HD + Bar groups markedly differed from the control ND, indicating that diet drives gut microbiota community structures. The HD diet was characterized by high fat and high simple sugars (sucrose), while the ND diet mice had high complex polysaccharides (corn starch and maltodextrin), which might have explained the higher level of saccharolytic fermentative gut bacteria, such as *Bacteroides* and *Ruminococcus*, in the ND groups compared to the HD. As previously documented [2], the mechanisms linking gut microbiota dysbiosis to metabolic diseases include enhanced energy harvesting from the diet, dysregulation of incretin signaling, metabolic endotoxemia and chronic systemic inflammation. Whether these mechanisms operate either in isolation or synergistically remains to be determined as does whether microbiota dysregulation is a consequence of metabolic disease or plays an etiological role in disease onset. Cytokine signaling via JAKs plays an important role in the pathogenesis of metabolic diseases as recently documented by both clinical and basic science research studies [6]. Following selective binding of cognate ligands, several receptors can recruit JAK proteins, leading to their dimerization and activation through autophosphorylation. Recent studies based on tissue-specific KO mice pointed out the essential role of the JAK-STAT cascade, mainly the JAK2 pathway, in the peripheral metabolic organs, including the adipose tissue, liver, skeletal muscle and pancreas. For instance, JAK2 deficiency in either hepatocytes, macrophages or adipocytes confers resistance to diet-induced metabolic stress and protects against HD-induced metaflammation and insulin resistance [[12], [13], [14]]. In contrast, loss of JAK3 in mice results in significant body weight increase, associated with impaired glycemic homeostasis, hyperinsulinemia and early symptoms of liver steatosis [11]. The JAK3 KO mice were more prone to developing high-fat induced metabolic syndrome than the control mice. Although these findings collectively demonstrate that activation of the JAK2 pathway does indeed occur in T2DM and may play a role in its pathogenesis, its potential as a pharmacological target for drug therapy of diet-induced metabolic derangements has been poorly investigated. In this study, we demonstrated for the first time that the chronic administration of baricitinib, an oral JAK1/2 inhibitor approved for the treatment of rheumatoid arthritis, protects against the deleterious effects of chronic high-fat high-sugar feeding. While the weight gain induced by HD was extremely rapid, more than 8 weeks of baricitinib administration were needed to detect a significant reduction, presumably because baricitinib treatment started after the HD mice had already developed obesity and insulin resistance (reversal study). Despite the observed changes in body weight gain induced by baricitinib, the HD and HD + Bar mice did not differ in gut microbiota composition, demonstrating the lack of direct local effects on gut bacteria when baricitinib is administered orally. These findings further support the theory that microbiota is involved, together with host physiological changes induced by the obesogenic diet, in triggering inflammation. However, recent evidence suggests that short-chain fatty acids (SCFAs) generated by gut microbiota have a wide range of functions from immune regulation to metabolism in a variety of tissues and organs and therefore may directly or indirectly regulate physiological and pathological processes in relation to obesity [35,36]. SCFAs have been demonstrated to participate in the mechanisms of insulin secretion from the pancreatic β cells and the release of peptide hormones that control appetite [37]. Thus, the lack of data on fecal content of SCFAs does not allow us to exclude that the beneficial effects of baricitinib are due (at least in part) to potential drug interactions with particular bacterial metabolites that may trigger regulatory metabolic cascades. In this study we mainly focused on the potential drug effects on target organs and tissues of diet-induced metabolic derangements. We demonstrated that baricitinib protects against the deleterious effects of HD by inhibiting the JAK-STAT pathway in the skeletal muscle, which exerts a key role in the regulation of glucose homeostasis. Among the best characterized activators of JAK signaling in the context of metabolic disorders, certain adipokines, including leptin and resistin, are known to be involved in the pathogenesis of insulin resistance [38,39]. The increase in their systemic concentrations evoked by chronic exposure to an hypercaloric diet, as confirmed herein, may contribute to the over-activation of JAK2 in both the skeletal muscle and kidneys of the HD mice. When activated, JAK phosphorylates STAT proteins, leading to their activation. Activated STAT proteins can dimerize and translocate to the nucleus where they regulate gene transcription, including cytokines production. Clinical data showed an increased phosphorylation of JAK2 and STAT in skeletal muscle biopsies from patients with T2DM and correlate with measures of insulin resistance [40,41]. In this study, we confirmed that HD does indeed drive the phosphorylation of JAK2 and STAT and reported that the JAK1/2 inhibitor baricitinib attenuated the phosphorylation of both JAK2 and STAT in mice with an HD. These effects of baricitinib were associated with a significant reduction in the plasma concentrations of the JAK-STAT dependent cytokines IL-1β, TNF-α, and IFN-γ, whose role in obesity and insulin resistance has been widely documented. In contrast, the anti-inflammatory cytokine IL-10 was upregulated following baricitinib exposure. We also documented a significant increase in IL-6 concentrations following baricitinib administration, consistent with a previous study showing that IL6-deficient mice fed HD develop significant hepatosteatosis and insulin resistance [42]. In fact, IL-6 is not a classical pro-inflammatory cytokine and may exert several anti-inflammatory actions, including downregulation of IFN-γ, IL-1β, and TNF-α [43]. Treatment of the HD mice with baricitinib also resulted in an increase in the plasma levels of GIP and ghrelin, both of which are involved in glucose homeostasis conferring protective effects for metabolic dysfunctions. In fact, beyond its insulinotropic effects in the pancreas, GIP has been demonstrated to exert important biological actions, including regulation of fat metabolism, with a key role in modulating lipid storage in adipose tissue [44]. Ghrelin signaling has been demonstrated to be another key mediator linking nutrient-sensing signals with adipose inflammation and insulin resistance. Ablation of ghrelin has been reported to worsen diet-induced obesity, insulin resistance and adipose inflammation [45].

Our study provided the first evidence that the improved glucose tolerance evoked by baricitinib administration in HD is, at least partially, mediated by enhancing the insulin-related signaling pathway in both the liver and skeletal muscle. The IRS-1/AKT/GSK-3β pathway is a crucial regulator of glucose transportation, glycogen synthesis and glycolysis [46]. The defects in insulin signaling observed in the liver and skeletal muscle of the HD fed mice were restored by pharmacological inhibition of JAK activity. Our data are agree with previously published papers reporting a cross-talk between the insulin and JAK signaling cascades [[47], [48], [49]]. In response to the administration of physiological concentrations of insulin in rats, JAK2 associates with the insulin receptor and is then phosphorylated in insulin-sensitive tissues [50]. Interestingly, JAK2 over-activation has been demonstrated to contribute to modulating AKT phosphorylation and glucose uptake in models of insulin resistance, whereas JAK2 knockdown in L6 myotubes alleviated insulin resistance by interfering with the negative regulation of AKT phosphorylation and activity [51]. Overall, our results, in line with previously published data, demonstrated that the activation of JAK2 signaling may directly modulate the AKT pathway, thus affecting a crucial pathogenic mechanism responsible for the development of insulin resistance. As documented in patients with rare molecular defects in the insulin signaling pathway [52], metabolic dyslipidemia is the result of selective post-receptor hepatic insulin resistance. Thus, we speculate that the preservation of insulin sensitivity may also account for improved systemic lipid profiles and the decrease in the deposition of intramuscular and perimuscular fat in the quadriceps muscle of the HD mice treated with baricitinib. Unfortunately, the lack of data on hepatic lipid levels does not allow us to conclude that the drug's beneficial effects on lipid profiles can be extended to the liver itself. We also documented significant inhibition of the JAK2-STAT2 pathway in the kidneys of the HD mice treated with baricitinib. Podocyte-specific JAK2 overexpression worsens diabetic kidney disease in mice [53]. This effect was associated with a significant reduction in HD-induced renal injury (histology) and proteinuria. In line with the reduction in proteinuria afforded by baricitinib, we recorded significant prevention of the expansion of mesangial cells and glomerular hypertrophy, both of which are hallmarks of diabetic nephropathy. These effects were due, at least in part, to baricitinib-induced reduction in cellular senescence as documented by the significant reduction in renal expression of the cyclin kinase inhibitor p21WAF1/CIP1 (p21). Hyperglycemia is known to evoke kidney cell senescence through a p21-dependent pathway. More specifically, high glucose via the JAK2 pathway enhances the expression of p21, which leads to cell cycle arrest in the G0/G1 phase and increased expression of extracellular matrix proteins such as fibronectin and type IV collagen and cellular hypertrophy of LLC-PK1 cells [54]. Thus, the reduced expression of p21 recorded in the kidneys of the HD mice exposed to baricitinib may be the direct consequence of JAK-STAT inhibition, that, along with the improvement in insulin sensitivity and plasma glucose levels, may contribute in direct and indirect manners to reducing mesangial cell expansion and proteinuria. Notably, these findings were consistent with a recently published phase 2 randomized controlled clinical trial that tested the efficacy of baricitinib in participants at a high risk of progression of diabetic kidney disease, demonstrating significant reductions in ACR over 24 weeks with baricitinib treatment [15]. We speculate that the cross-talk mechanism of action investigated contributed, at least in part, to reaching the primary trial endpoint.

## 5 Conclusion

In conclusion, our results support the view that activation of the JAK2-STAT2 pathway drives the development of obesity, insulin resistance, associated end-organ injury and, most notably, highlights the potential repurposing of JAK inhibitors already approved for rheumatoid arthritis as promising treatment options in the clinical context of metabolic diseases. In fact, the use of the JAK 1/2 inhibitor baricitinib, which was administered for the last 16 weeks of the 22-week dietary manipulation, exerted multi-organ protection against the deleterious effects of HD exposure. Interestingly, targeting metaflammation by inhibiting JAK signaling by orally administered small molecules such as baricitinib might offer several advantages compared with a therapeutic strategy centered on targeting cytokines with biologics, including better pharmacokinetics and cost effectiveness, fewer immunosuppressive effects and wider simultaneous targeting of multiple pathogenic pathways, making them very attractive and potentially suitable for drug repurposing.

## Author contributions

Christoph Thiemermann and Massimo Collino: Conceptualization, supervision, writing, reviewing, and editing. Debora Collotta and William Hull: Investigation, data curation, writing and original draft preparation. Raffaella Mastrocola, Fausto Chiazza, Alessia Sofia Cento, Catherine Murphy, Roberta Verta, Gustavo Ferreira Alves and Giulia Gaudioso: Investigation, data collection and analysis. Francesca Fava, Manuela Aragno and Kieran Tuohy: Supervision, validation, critical review and revision. All of the authors revised the manuscript and approved the final version.

## Funding

This study was supported by the Italian Ministry of Agricultural, Alimentary, and Forestry Policies (grant ID: 777 HDHL INTIMIC-Knowledge Platform, grant ID: 1170 HDHL INTIMIC METADIS and grant ID: CABALA_diet&health, ERA HDHL), the Italian Ministry of Education, University, and Research (grant ID: SALIVAGES, ERA HDHL, and grant ID: CABALA_diet&health, ERA HDHL), the 10.13039/501100006692University of Turin (grant ID: Ricerca Locale Ex-60%), the 10.13039/501100000268Biotechnology and Biological Sciences Research Council (BBSRC) UK with Takeda Cambridge Pharmaceuticals Ltd. (award reference 1507427), and the Center for Diabetic Kidney Disease (Barts & London Charity).
